# Supplementary material for: Causal interplay between lactose intolerance and gut microbiota: a combined bidirectional Mendelian randomization and in vivo validation study
Source: Front Nutr. 2026 Jun 1;13:1803337. doi: 10.3389/fnut.2026.1803337 (PMC13265576; doi:10.3389/fnut.2026.1803337)
Supplement: Supplementary file 1 [file Data_Sheet_1.zip › supplementary materials/Forward/leave one out cross validation/ebi-a-GCST90027495.finngen_R12_E4_LACTONAS.pdf]

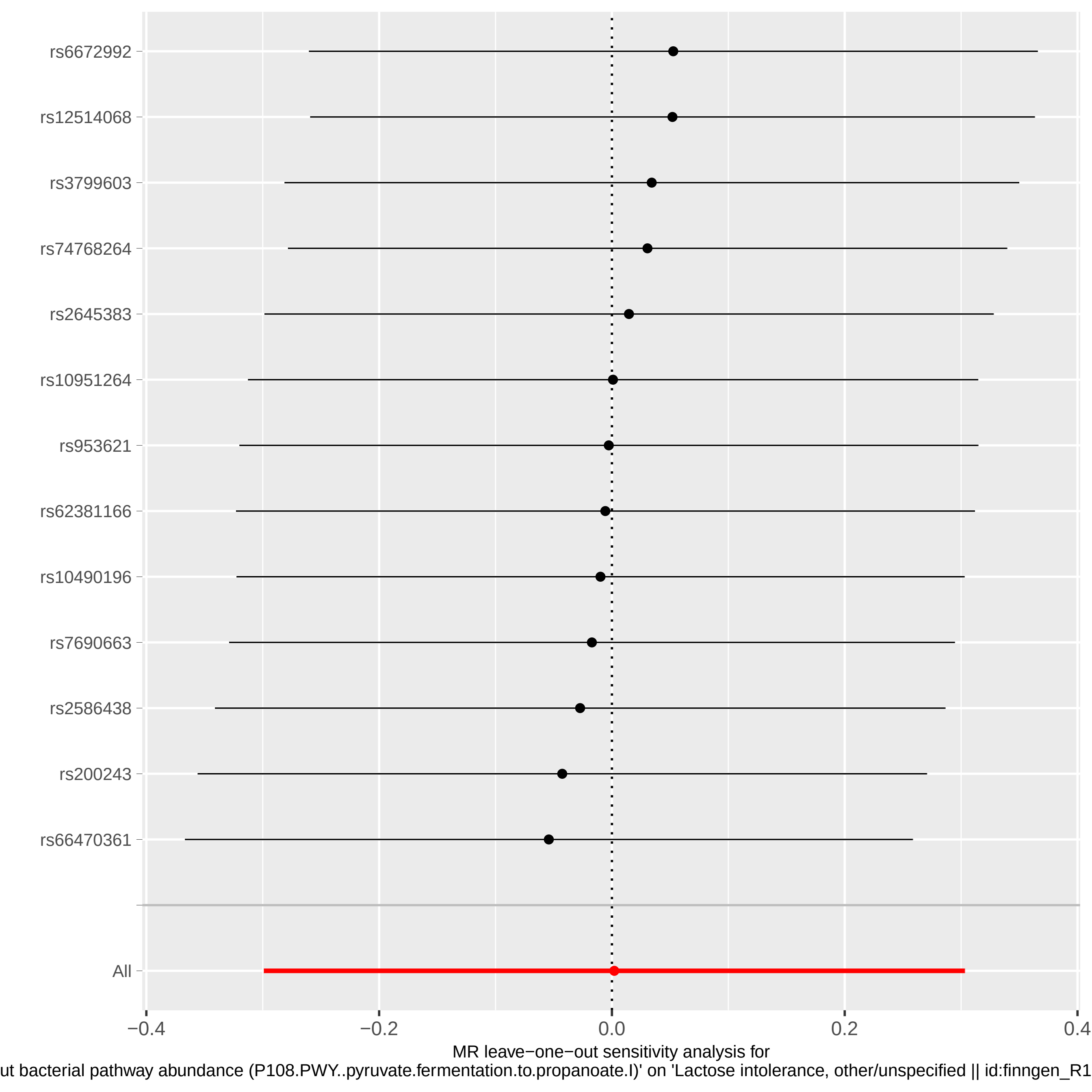
rs6672992
rs12514068
rs3799603
rs74768264
rs2645383
rs10951264
rs953621
rs62381166
rs10490196
rs7690663
rs2586438
rs200243
rs66470361
All
−0.4
−0.2
0.0
0.2
0.4
MR leave−one−out sensitivity analysis for
ut bacterial pathway abundance (P108.PWY..pyruvate.fermentation.to.propanoate.I)' on 'Lactose intolerance, other/unspecified || id:finngen_R1
